# Insulin-Like Growth Factor 1 in the Preterm Rabbit Pup: Characterization of Cerebrovascular Maturation following Administration of Recombinant Human Insulin-Like Growth Factor 1/Insulin-Like Growth Factor 1-Binding Protein 3

**DOI:** 10.1159/000516665

**Published:** 2021-07-02

**Authors:** Magnus Gram, Claes Ekström, Bo Holmqvist, Galen Carey, Xiaoyang Wang, Suvi Vallius, William Hellström, Niklas Ortenlöf, Alex Adusei Agyemang, Lois E.H. Smith, Ann Hellström, Alexandra Mangili, Norman Barton, David Ley

**Affiliations:** a Department of Clinical Sciences Lund, Pediatrics, Lund University, Lund, Sweden; b ImaGene-iT AB, Lund, Sweden; c Takeda Pharmaceuticals, Boston, MA, USA; d Institute of Neuroscience and Physiology, Sahlgrenska Academy, University of Gothenburg, Gothenburg, Sweden; e Department of Pediatrics, Institute of Clinical Sciences, Sahlgrenska Academy, University of Gothenburg, Gothenburg, Sweden; f Department of Ophthalmology, Boston Children’s Hospital, Harvard Medical School, Boston, MA, USA; g Department of Clinical Neuroscience, Sahlgrenska Academy, University of Gothenburg, Gothenburg, Sweden; h Global Clinical Development, Rare Metabolic Diseases, Shire, a Takeda Company, Zurich, Switzerland

**Keywords:** Brain development, Insulin-like growth factor-1 system, Intraventricular hemorrhage, Prematurity, Vascular development

## Abstract

Following preterm birth, serum levels of insulin-like growth factor 1 (IGF-1) decrease compared to corresponding in utero levels. A recent clinical trial indicated that supplementation with recombinant human (rh) IGF-1/rhIGF-binding protein 3 (rhIGF-1/rhIGFBP-3) prevents severe intraventricular hemorrhage (IVH) in extremely preterm infants. In a preterm rabbit pup model, we characterized endogenous serum and hepatic IGF-1, along with brain distribution of IGF-1 and IGF-1 receptor (IGF1R). We then evaluated the effects of rhIGF-1/rhIGFBP-3 on gene expression of regulators of cerebrovascular maturation and structure. Similar to preterm infants, serum IGF-1 concentrations decreased rapidly after preterm birth in the rabbit pup. Administration of rhIGF-1/rhIGFBP-3 restored in utero serum levels but was rapidly eliminated. Immunolabeled IGF1R was widely distributed in multiple brain regions, displaying an abundant density in the choroid plexus and sub-ependymal germinal zones. Increased IGF-1 immunoreactivity, distributed as IGF1R, was detected 4 h after rhIGF-1/rhIGFBP-3 administration. The rhIGF-1/rhIGFBP-3 treatment led to upregulation of choroid plexus genes involved in vascular maturation and structure, with corresponding protein translation for most of these genes. The preterm rabbit pup model is well suited for evaluation of IGF-1-based prevention of IVH. Administration of rhIGF-1/rhIGFBP-3 affects cerebrovascular maturation, suggesting a role for it in preventing preterm IVH.

## Introduction

Insulin-like growth factor-1 (IGF-1) is a mitogenic factor involved in metabolism and angiogenesis, with an important role in fetal development [1]. Following preterm birth, serum levels of IGF-1 decrease and are substantially lower than those in fetuses of corresponding gestational age (reviewed in [2]). Low postnatal IGF-1 serum levels are associated with an increased risk of neurodevelopmental impairment [3, 4]. Most of the physiological effects of IGF-1, such as cell proliferation, growth, and differentiation, are mediated via the cell surface receptor IGF-1 receptor (IGF1R) [5] and the canonical phosphatidylinositol 3-kinase (PI3K)/Akt and Ras/extracellular signal-regulated kinase (ERK) signaling pathways [6, 7]. The biological activity and biodistribution of circulatory IGF-1 are largely regulated through complex formation with specific carrier proteins, the IGF-binding proteins (IGFBPs) 1–6, of which IGFBP-3 is most abundant, representing approximately 75–80% of the total circulatory IGF-1 pool. In circulation, IGF-1, IGFBP-3, and an acid-labile subunit form a ternary complex of approximately 150–200 kDa that substantially extends the serum half-life of IGF-1 [8].

Significant improvement in neonatal care over the last decades has enabled increased survival among infants who are born extremely preterm (EPT) [9]. However, major neonatal morbidities such as bronchopulmonary dysplasia, retinopathy of prematurity, intraventricular hemorrhage (IVH), and necrotizing enterocolitis are highly prevalent and often associated with neurodevelopmental disability or mortality [10, 11].

About 35% of EPT infants with a very low birth weight are affected by some degree of cerebral IVH, and approximately 16% of IVH lesions are severe, corresponding to an IVH grade 3 or 4 [12]. More than 50% of infants with severe IVH develop posthemorrhagic ventricular dilatation, and 35% experience severe neurological impairment, mainly cerebral palsy [13]. The vast majority of all hemorrhages, around 80%, occur within the first 72 h after birth, with a considerable proportion arising within the first 6 h [14, 15]. No approved therapy is available to prevent or treat IVH in EPT infants.

In a recent phase 2 randomized controlled trial, treatment of infants born at gestational age 23–27 weeks with a continuous intravenous (i.v.) infusion of recombinant human (rh) IGF-1/rhIGFBP-3 (250 μg/kg/24 h) resulted in a trend toward decreased occurrence of severe IVH as compared with standard neonatal care (8.3 vs. 23.3% in the evaluable set; not significant) [16]. Although findings from several observational studies have suggested that supplementary treatment with IGF-1, targeting serum concentrations within the normal intrauterine range, would improve growth and reduce neonatal morbidities, including IVH [1], the causal mechanisms underlying any treatment effect are unknown. Current understanding of the etiology of IVH is that it arises because of an inherent fragility of the germinal matrix vasculature resulting from an immature basal lamina, a scarcity of pericytes, and a high rate of endothelial proliferation [17–19]. Based on this understanding, we hypothesized that treatment with rhIGF-1/rhIGFBP-3 would decrease vascular fragility and vulnerability to fluctuations in cerebral blood flow by increasing expression of structural vascular components and factors inducing vascular maturation.

In this study, we used the preterm rabbit pup model to evaluate the effects of rhIGF-1/rhIGFBP-3 on regulators of cerebrovascular maturation and structure. This model is well suited for studies of IGF-1 related effects on prematurity as well as preterm IVH because it incorporates essential physiological aspects of human preterm birth, including an abrupt decrease in endogenous circulating IGF-1 levels [20]. In addition, induction of IVH in the preterm rabbit pup by a hyperosmolar insult through intraperitoneal administration of glycerol triggers a series of events that largely resembles what occurs in preterm infants. These events include perturbations of cerebral blood flow exerting mechanical stress on the structural integrity of the vessels, leading to a subsequent rupture and bleeding into the ventricles [21]. In a recent study, Ley et al. [22] showed that the hemorrhage primarily affects the ventricles, choroid plexus, subfornical organ, subventricular zone, and to some extent also the hippocampus and dorsal thalamus. Here, we investigated the rhIGF-1/rhIGFBP-3 serum pharmacokinetic (PK) profile, distribution of IGF-1 and IGF1R in the brain, and the effects of rhIGF-1/rhIGFBP-3 on expression of genes and proteins involved in angiogenesis and extracellular matrix (ECM) structure in the choroid plexus of preterm rabbit pups.

## Materials and Methods

We used several experimental setups in the current studies. All details of each setup are given in the online suppl. material; see www.karger.com/doi/10.1159/000516665 for all online suppl. material, online suppl. Tables 1 and 2, and online suppl. Figures 1 and 2. Here, we provide a brief summary.

### Animals

The animal protocols were approved by the Swedish Animal Ethics Committee in Lund, Sweden. Reporting is in compliance with the ARRIVE guidelines. We used the well-established preterm rabbit pup model in accordance with previous description [21]. A half-breed between New Zealand White and Lop was used. Briefly, the experiments were performed on a total of 306 rabbit pups from 55 litters (168 females and 138 males; for details, see online suppl. Table 1) delivered on day 29 (term 31–32 days) via cesarean section, after the does were anesthetized. After delivery, the pups were placed in an infant incubator set to 30°C and 60% humidity. In a sub-study (see experimental setup below and online suppl. material for more details), rabbit pups were delivered vaginally at term (31–32 days).

### Experimental Setup: Characterization of IGF-1, IGF1R, and Cerebrovascular Maturation

Following birth, the cesarean-delivered preterm rabbit pups were handled and nursed by the animal laboratory staff, whereas the does nursed the vaginally delivered term rabbit pups. Pups were randomized to experimental groups, treatment regimens, and termination time points. In some experimental groups, rhIGF-1/rhIGFBP-3 or the corresponding vehicle was administered subcutaneously (s.c.) once or repeatedly at doses of 1.0–8.0 mg/kg/dose (at a fixed volume of 0.1 mL/animal/dose). This dosing was started in animals at either approximately 3 h of age and repeated every 12 h up to an age of approximately 72 h (1–7 administrations of rhIGF-1/rhIGFBP-3 or vehicle in total) or starting at an age of approximately 72 h and followed up to an age of approximately 120 h (1 administration of rhIGF-1/IGFBP-3 or vehicle in total). Pups were terminated at the endpoint determined for each experimental group, ranging from 0 to 120 h of age, and samples of one or more of the following were collected and analyzed as described below: blood, used for determination of serum IGF-1 levels; liver biopsy, used for IGF-1 gene expression analysis; brain, used for IGF-1, IGF1R, and cerebrovascular immunolabeling; choroid plexus, used for ECM- and angiogenesis-related gene expression analysis; and ear biopsy, used for sex determination.

### Tissue Collection and Processing for Histology and Immunolabeling

Brain tissue was prepared as described in the online suppl. material. Briefly, the rabbit pups were transcardially perfused, and the brains excised and immersion fixed in freshly prepared 4% paraformaldehyde. Subsequently, the brain tissue was prepared and sectioned into coronal and sagittal sections (4 or 6 μm).

### Immunohistochemistry

Tissue preparations and sections were prepared and evaluated as described in the online suppl. material. Briefly, labeling of IGF1R and IGF-1 was performed using a primary polyclonal (IGF1R) or monoclonal (IGF-1) antibody followed by a horseradish peroxidase-conjugated secondary antibody, and a diaminobenzidine reaction was used to visualize the immunoreactive sites. The IGF1R distribution was evaluated by visual inspection under a bright-field microscope and in digital images of slide scanned whole sections. Color-coded images of IGF1R immunoreactivity were made to illustrate the differentiated IGF1R densities in the brain.

### Immunofluorescence

For details on tissue preparation, immunofluorescence procedure, and evaluation, see the online suppl. material. Briefly, single- or double-immunofluorescence labeling was performed to evaluate the detailed distribution of IGF1R and establish the relation between IGF1R versus IGF-1 and IGF-1 versus endothelial cells (CD31). Immunofluorescence labeling was also used to detect and visualize the proteins, indicated as influenced by rhIGF-1/rhIGFBP-3 administration in the gene expression analysis, using primary antibodies against angiopoietin-1 (ANGPT1), fibronectin-1 (FN1), procollagen-1 (PROCOL), versican (VCAN), and thrombospondin-1 (THBS1). Sections were incubated with the primary antibodies or an antibody combination followed by incubation with 1 or 2 fluorophore-conjugated secondary antibodies. Sections were then counterstained with 4′,6-diamidino-2-phenylindole. Analysis and imaging were performed using a confocal laser scanning microscope. For digital quantitation of the labeled proteins, we imaged the immunofluorescence labeling of ANGPT1, FN1, PROCOL, VCAN, and THBS1 in selected regions of interest, that is, the choroid plexus, subfornical organ, subarachnoid space, capillaries, and periventricular region. To quantify the target proteins, we calculated a ratio of the labeled area (for a given protein) per cell nucleus area.

### Tissue Collection and Processing for mRNA

Rabbit pups were euthanized at 0–72 h after rhIGF-1/rhIGFBP-3 or vehicle administration, and the brain tissue was harvested for choroid plexus gene expression analysis as described in the online suppl. material. In addition, a liver biopsy sample was collected, snap frozen, and stored at −80°C for mRNA analysis, as described below.

### RNA Isolation, Gene Array, and Real-Time PCR

Total RNA from liver and choroid plexus tissue was extracted and analyzed as described in the online suppl. material. Briefly, total RNA was isolated, and reverse transcription was performed with the RT2 First Strand Kit and iScript cDNA Synthesis Kit according to the manufacturer. The expression of 84 genes related to ECM and adhesion molecules (RT2 Profiler PCR Array) and angiogenesis (RT2 Profiler PCR Array) were evaluated using either pooled (4 and 72 h) or individual (24 h) choroid plexus tissue samples from respective group. Data were normalized to glyceraldehyde-3-phosphate dehydrogenase, and the fold change values were calculated by normalizing data against representative control samples from vehicle-treated animals (see Fig. 4 and 5 for details). Data were visualized as fold change in a heat map, and the most significantly altered genes (i.e., up- or downregulated more than 2-fold) at the respective time points are listed. Based on the outcome of the profiling arrays, the significantly altered genes collagen type I alpha 1 (COL1A1), IGF-1, FN1, VCAN, THBS1, and ANGPT1 were further evaluated in specific qPCR analysis using the RT2 Primer assay. Data were normalized against glyceraldehyde-3-phosphate dehydrogenase, and the fold change values were calculated by normalizing data against representative control samples from vehicle-treated animals (see Fig. 6 for details). Data were visualized as fold change at the respective time points.

### Serum IGF-1 Concentrations

Serum concentration of IGF-1 was determined using the Human IGF-1 ELISA (Mediagnost, Reutlingen, Germany). The manufacturer provided the information that this assay is applicable for rabbit serum samples. The analysis was performed according to the manufacturer’s instructions.

### Sex Determination

Rabbit sex was determined by confirming the presence of the sex determining region Y gene (gene ID: 100328958) of the rabbit genome, using PCR and gel electrophoresis visualization, as described in the online suppl. material.

### Statistics

Statistical significance was calculated with a 1-way ANOVA corrected for multiple comparisons (Bonferroni or Sidak). Fold change regulation, comparing treatment group versus control group, was evaluated using Student’s *t* tests. *p* values <0.05 were considered significant. Statistical analyses were performed using GraphPad Prism (GraphPad Prism 8; GraphPad).

## Results

### Description of the Animals

Overall, 405 rabbit pups were delivered from 55 pregnant does, and 306 (76%) were included in the studies, of which 300 (98%) were preterm (cesarean section delivery at gestational day E29). Of the 99 (24%) pups excluded from the analysis, and thus not included in the evaluated study material, approximately 10–15% died before randomization to any study group, and the remainder died before scheduled termination (online suppl. Table 1). The overall sex distribution was fairly balanced at 168 females (55%) and 138 males (45%), although with a somewhat larger variation within the individual litters (online suppl. Table 1). The body weight of preterm (E29 + 0 h) pups at birth (median 40 g, range 24–58 g) was lower than that of term (P0 + 0 h) pups (median 54 g, range 49–56 g; *p* < 0.001) and remained low (median 40–43 g, range 24–61 g) throughout the study period (E29 + 0 h to E29 + 120 h) (online suppl. Table 2). Head circumference at birth of preterm pups (median 16 mm, range 13–21 mm) was lower than that of the term pups (median 20 mm, range 19–20 mm; *p* < 0.001) (online suppl. Table 2).

### Endogenous Serum Protein Levels and Hepatic mRNA Expression of IGF-1

**Fig. 1.**
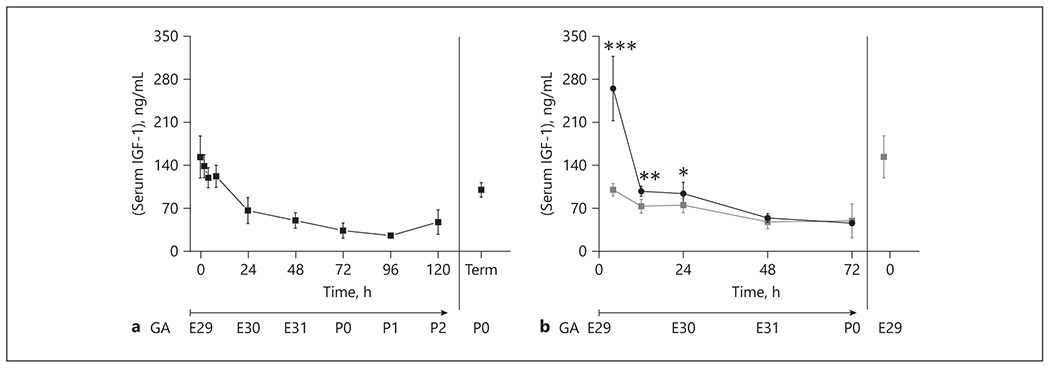
Serum IGF-1. **a** Endogenous serum IGF-1 protein level was determined in preterm rabbit pups at 0–120 h postnatal age (corresponding GA is included on the *x*-axis) and in term rabbit pups, vaginally delivered at P0, as described in the Material and Methods. **b** Preterm rabbit pups were dosed s.c. with 8 mg/kg/dose of rhIGF-1/rhIGFBP-3 (black circles) or corresponding vehicle (grey squares) from 3 h of age and every 12 h until 75 h of age (for a total of 1–7 administrations depending on the time of termination; see Material and Methods for details). Blood samples were collected at 4, 12, 24, 48, and 72 h after the first dosing, and serum IGF-1 was analyzed as described in the Materials and Methods. Corresponding serum IGF-1 levels of preterm rabbit pups at birth (E29) are shown as grey squares. Results are presented as mean ± SD. Differences between rhIGF-1/rhIGFBP-3 versus vehicle-treated animals at the respective time points were analyzed using 1-way ANOVA with post hoc Bonferroni correction. *p < 0.05, **p < 0.01, ***p < 0.001. GA, gestational age; E, embryonic; P, postnatal; IGF-1, insulin-like growth factor 1

The endogenous IGF-1 serum protein level was determined in preterm and term pups at 0–120 h postnatal age. The IGF-1 serum protein concentration decreased rapidly following preterm birth (median 149.8 ng/mL, range 93.1–220.4 ng/mL at E29 + 0 h) and was significantly lower by 4 h of age (median 123.0 ng/mL, range 81.3–135.5 ng/mL at E29 + 4 h; *p* < 0.01), reaching a trough at a postnatal age of 72 h (median 31.5 ng/mL, range 19.8–69.0 ng/mL at E29 + 72 h) that remained low throughout the study (median 48.1 ng/mL, range 23.2–76.0 ng/mL at E29 + 120 h) (Fig. 1a; online suppl. Table 3). Furthermore, the serum IGF-1 protein level of preterm pups at a postnatal age corresponding to the P0 term equivalent age (E29 + 72 h; median 31.5 ng/mL, range 19.8–69.0 ng/mL) was significantly lower than the IGF-1 serum concentration of term pups at P0 + 0 h (median 101.8 ng/mL, range 81.0–111.2 ng/mL; *p* < 0.001) (Fig. 1a; online suppl. Table 3).

Hepatic IGF-1 mRNA expression was characterized in preterm and term pups at several of the time points corresponding to those of serum IGF-1 protein levels. Overall, the hepatic expression of IGF-1 in preterm rabbit pups was relatively stable during the first 120 h (online suppl. Table 3). A small but significant increase was observed at a postnatal age of 24 h (approximately 2.4-fold, E29 + 24 h) and 120 h (approximately 3.9-fold, E29 + 120 h) as compared to expression at birth (E29 + 0 h) (online suppl. Table 3). The trend toward an increase between 24 and 120 h, that is, a fold change increase from 2.4 to 3.9, was not statistically significant (*p* = 0.453). This was further supported by an unaltered expression at 72 h as compared to the levels at birth (online suppl. Table 3). Comparison of IGF-1 mRNA expression between preterm and term rabbit pups showed a small but significantly higher level in pups delivered at term (P0 + 0 h: approximately 2.1-fold; *p* < 0.001) as compared to preterm pups at birth (E29 + 0 h). In contrast, no significant difference was observed when comparing term (P0 + 0 h) with preterm pups at 72 h of postnatal age, corresponding to P0 term equivalent age (E29 + 72 h) (online suppl. Table 3).

### Characterization of IGF1R in the Newborn Rabbit Brain

**Fig. 2.**
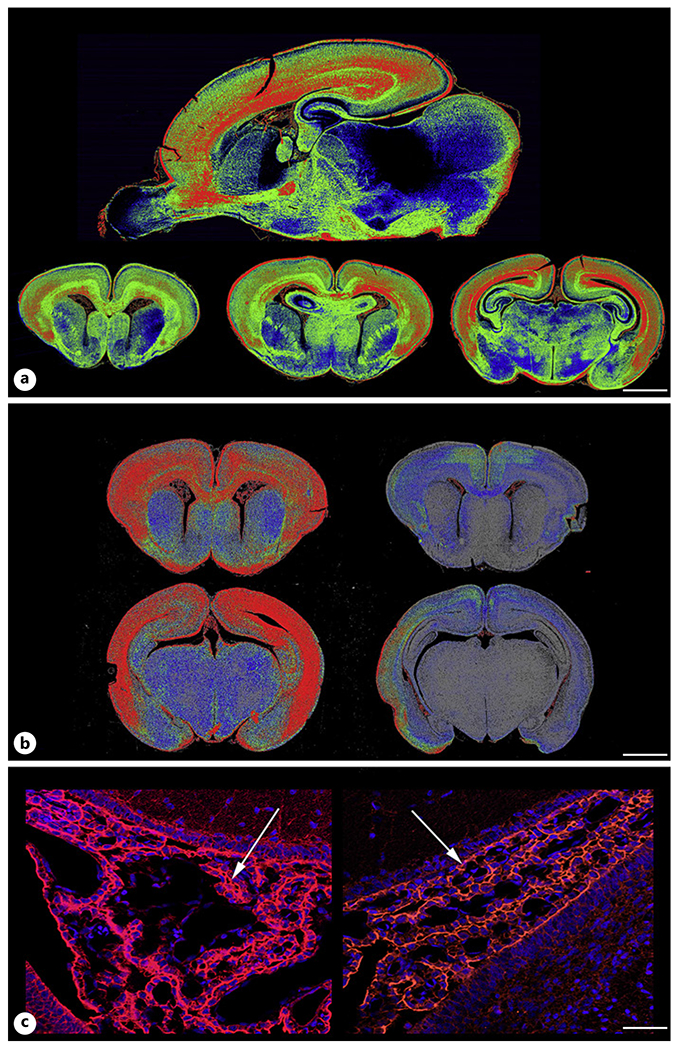
IGF1R immunoreactivity. **a** Color-coded images illustrate the differentiated density of IGF1R immunoreactivity in whole brain sections from E29 preterm pups. Representative brain sections, sagittal view in top section and coronal view in bottom section, demonstrate the IGF1R density color-coded as high (red), medium (green), and low (blue) intensity. A high density of IGF1R was detected in the choroid plexus, subfornical organ, periventricular regions, and major fiber tracts. **b** Images illustrate representative coronal sections of the preterm brain at birth (left panel, E29 + 0 h) and at postnatal day 4 (right sections, E29 + 96 h). Congruent with the immunoreactivity in (**a**), the IGF1R density at E29 (the left panel) showed a high intensity, whereas a relatively low IGF1R density was seen at postnatal day 4 (right coronal sections). The color-coded IGF1R densities of sections in (**b**) are set at a different threshold value than in (**a**). **c** Confocal microscope images illustrate the detailed IGF1R localization in the choroid plexus of an E29 preterm rabbit pup, with pronounced labeling of the epithelial cells (arrows). IGF1R was observed on both the apical and basolateral sides of the endothelial-epithelial blood-cerebrospinal fluid barrier. IGF1R immunoreactivity was also observed in periventricular cell layers surrounding the ventricle. Cell nuclei were counterstained with DAPI. Scale bar in (**a**) represents 2 mm for the sagittal sections and 4 mm for the coronal sections. Scale bar in (**b**) represents 3 mm. Scale bar in (**c**) represents 50 μm. IGF1R, insulin-like growth factor 1 receptor; DAPI, 4′,6-diamidino-2-phenylindole.

Immunohistochemistry together with digital image analysis and immunofluorescence labeling for confocal microscopy analysis were used to characterize the distribution of the IGF1R in the newborn rabbit brain (Fig. 2a). IGF1R immunolabeling was widely distributed in the brain. Brain regions with the highest IGF1R densities included the choroid plexus, subfornical organ, meninges, periventricular regions, and major fiber tracts. No detectable difference in either distribution or density was observed between preterm and term pups (not shown). IGF1R was highly abundant at birth in both preterm (E29) and term pups (P0) but decreased with postnatal age (as illustrated in preterm pups at postnatal age E29 + 96 h) (Fig. 2b). Confocal microscopy analysis showed that the IGF1R labeling was mainly localized to outer cell membranes of cell bodies and along nerve fibers. In the choroid plexus, IGF1R labeling was detected on both the apical and basolateral sides of the endothelial-epithelial blood-cerebrospinal fluid barrier, with a more pronounced labeling of the epithelial cell layer (Fig. 2c).

### IGF-1 in Preterm Rabbit Pups following s.c. Administration of rhIGF-1/rhIGFBP-3

Following administration of one (1) s.c. dose of rhIGF-1/rhIGFBP-3 (1.0—8.0 mg/kg) or vehicle to preterm pups, given at a postnatal age of 72 h, we analyzed the serum IGF-1 protein levels. This time point was selected for start of dosing because it was when the lowest postnatal endogenous circulatory IGF-1 levels were observed, thus enabling a more comprehensive understanding of the PK of exogenously supplemented IGF-1. We observed a clear dose-dependent increase in serum IGF-1, peaking between 1 and 4 h post-administration and returning to baseline at approximately 24–48 h (online suppl. Table 4). Administration of a dose of 8 mg/kg resulted in peak IGF-1 serum levels that corresponded to endogenous levels at birth, that is, corresponding to IGF-1 levels in utero (153.4–181.5 ng/mL) (online suppl. Table 4); thus, we selected this dose for further studies. Based on data from the dose range finding study, a follow-up dosing study was performed using the 8 mg/kg/dose, administered from 3 h of age and every 12 h until 75 h of age (for a total of 1–7 administrations depending on the time of termination; see online suppl. material and methods for details). Blood samples were collected at 4, 12, 24, 48, and 72 h after the first dosing, and serum IGF-1 levels were analyzed. Of note, blood samples were collected at 12 h, or at 4 h for the 4-h sampling point, after the most recent administration, and just prior to the upcoming dosing at the respective time points.

The results showed a circulatory IGF-1 peak at approximately 4 h after s.c. administration of rhIGF-1/rhIGFBP-3 and restoration of serum IGF-1 levels that were similar to those observed at birth/in utero (E29 + 0 h) (Fig. 1b). Subsequently, levels of IGF-1 decreased rapidly, reaching values similar to those observed in vehicle-treated animals (Fig. 1b). However, at the age of 12 h (median IGF-1 of 94.6 ng/mL, *p* < 0.01) and 24 h (median IGF-1 of 96.0 ng/mL, *p* < 0.05), IGF-1 levels of animals exposed to the 8 mg/kg/dose of rhIGF-1/rhIGFBP-3 remained significantly higher than those in vehicle-treated animals.

Analysis of hepatic IGF-1 mRNA expression following s.c. administration of rhIGF-1/rhIGFBP-3 (1.0–8.0 mg/kg) displayed no apparent trends toward increased or decreased expression (online suppl. Table 5). However, despite the absence of a significant dose-response, there was a numerical increase in hepatic expression following exposure to 4 and 8 mg/kg/dose.

**Fig. 3.**
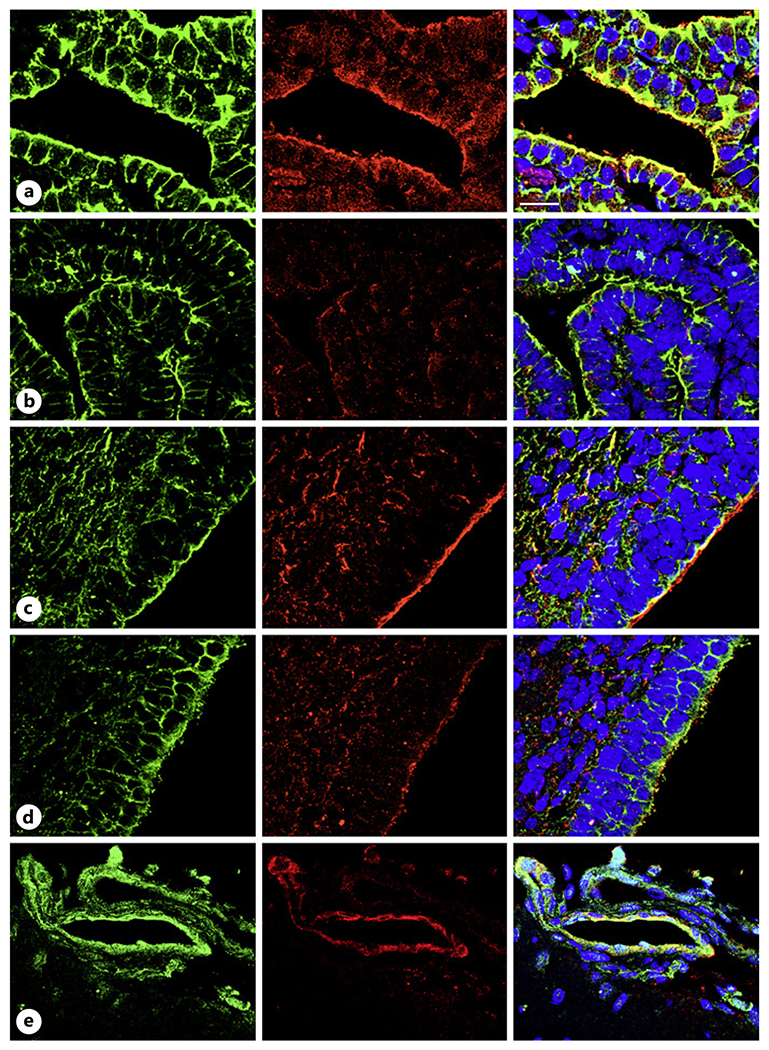
Distribution of IGF-1, IGF1R, and CD31. The distribution of IGF-1 (red) and its relation to IGF1R (green) was investigated in the brains of preterm rabbit pups at 4 h post s.c. administration of 8 mg/kg rhIGF-1/rhIGFBP-3 (**a**, **c**, **e**) and in vehicle-treated animals (**b**, **d**) by means of double immunofluorescence and confocal microscope analysis. Regions with high IGF1R densities (green, see also Fig. 2), the choroid plexus (illustrated in **a**, **b**), periventricular regions (illustrated in **c**, **d**), and vascular units (illustrated in **e**) displayed a corresponding high IGF-1 labeling (red) and indicated coexistence (**a–d**, yellow). In addition, IGF-1 labeling intensities were relatively higher in animals exposed to the 8 mg/kg dose of rhIGF-1/rhIGFBP-3 (**a**, **c**) compared to the IGF-1 labeling in vehicle-treated animals (**b**, **d**). **e** IGF-1 (red) was also detected at high levels in vascular units, illustrated by IGF-1 double labeled with CD31 (green) in blood vessels of the subarachnoid region. Cell nuclei were counterstained with DAPI. Scale bar in (**a**) represents 20 μm and is representative for (**a–e**). IGF-1, insulin-like growth factor 1; IGF1R, insulin-like growth factor 1 receptor; rhIGF-1, recombinant human insulin-like growth factor 1; rhIGFBP-3, recombinant human insulin-like growth factor binding protein 3; DAPI,4′,6-diamidino-2-phenylindole.

The distribution of IGF-1 within the immature brain was evaluated at 4 h post s.c. administration of 8 mg/kg rhIGF-1/rhIGFBP-3 or vehicle solution. Based on the IGF1R distribution (Fig. 2), for co-analysis of IGF-1, we selected the choroid plexus, subfornical organ, and periventricular regions, all of which showed high IGF1R densities (Fig. 3a–d). IGF-1 labeling was present in all regions of interest, with a corresponding distribution and structural coexistence with IGF1R (Fig. 3a–d, yellow). In all evaluated regions, relatively higher intensities of IGF-1 immunofluorescence were detected in animals exposed to 8 mg/kg rhIGF-1/rhIGFBP-3 (Fig. 3a, c) as compared to the vehicle-treated animals (Fig. 3b, d). Double immunolabeling for IGF-1 and an endothelial cell marker (CD31) showed a high IGF-1 content also in vascular units (Fig. 3e).

### rhIGF-1/rhIGFBP-3-Induced Increases in Expression of Genes Related to Angiogenesis, ECM, and Adhesion in the Choroid Plexus

**Fig. 4.**
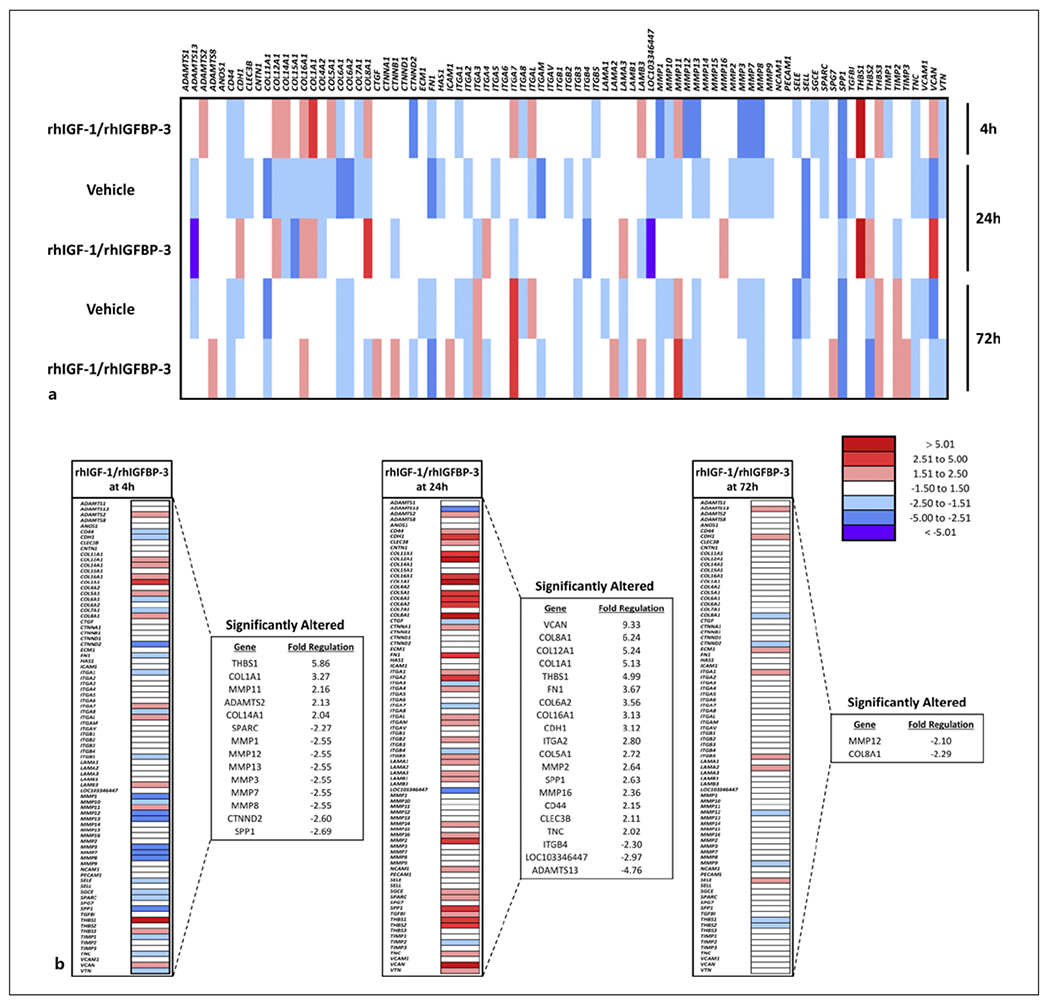
ECM and adhesion molecule array. The expression of 84 genes related to ECM and adhesion molecules was evaluated using RT2 Profiler PCR Arrays in choroid plexus tissue samples from preterm rabbit pups at 4, 24, and 72 h after exposure to 8 mg/kg/dose rhIGF-1/rhIGFBP-3 or vehicle. Animals were given rhIGF-1/rhIGFBP-3 or vehicle from 3 h of age and every 12 h until 75 h of age (for a total of 1–7 administrations depending on the time of termination). Analysis was performed on pooled samples from pups terminated at 4 h (*n* = 6 in the vehicle group and *n* = 6 in the rhIGF-1/rhIGFBP-3 group) and 72 h (*n* = 7 in the vehicle group and *n* = 8 in the rhIGF-1/rhIGFBP-3 group) after first dosing or individual samples from pups terminated at 24 h (*n* = 6 in the vehicle group and *n* = 7 in the rhIGF-1/rhIGFBP-3 group) after first dosing. **a** Data were normalized to GAPDH, included in the RT2 Profiler PCR Arrays, and the fold change values were calculated by normalizing against those of vehicle-treated animals at 4 h. Data are visualized as fold change in a heat map. **b** Data were normalized to GAPDH, included in the RT2 Profiler PCR Arrays, and the fold change values were calculated by normalizing against the vehicle-treated group at the respective time points. Data are visualized as fold change in a heat map, and the most significantly altered genes (i.e., up- or downregulated more than 2-fold) at the respective time points are listed. ECM, extracellular matrix; rhIGF-1, recombinant human insulin-like growth factor 1; rhIGFBP-3, recombinant human insulin-like growth factor binding protein 3; GAPDH, glyceraldehyde-3-phosphate dehydrogenase.

**Fig. 5.**
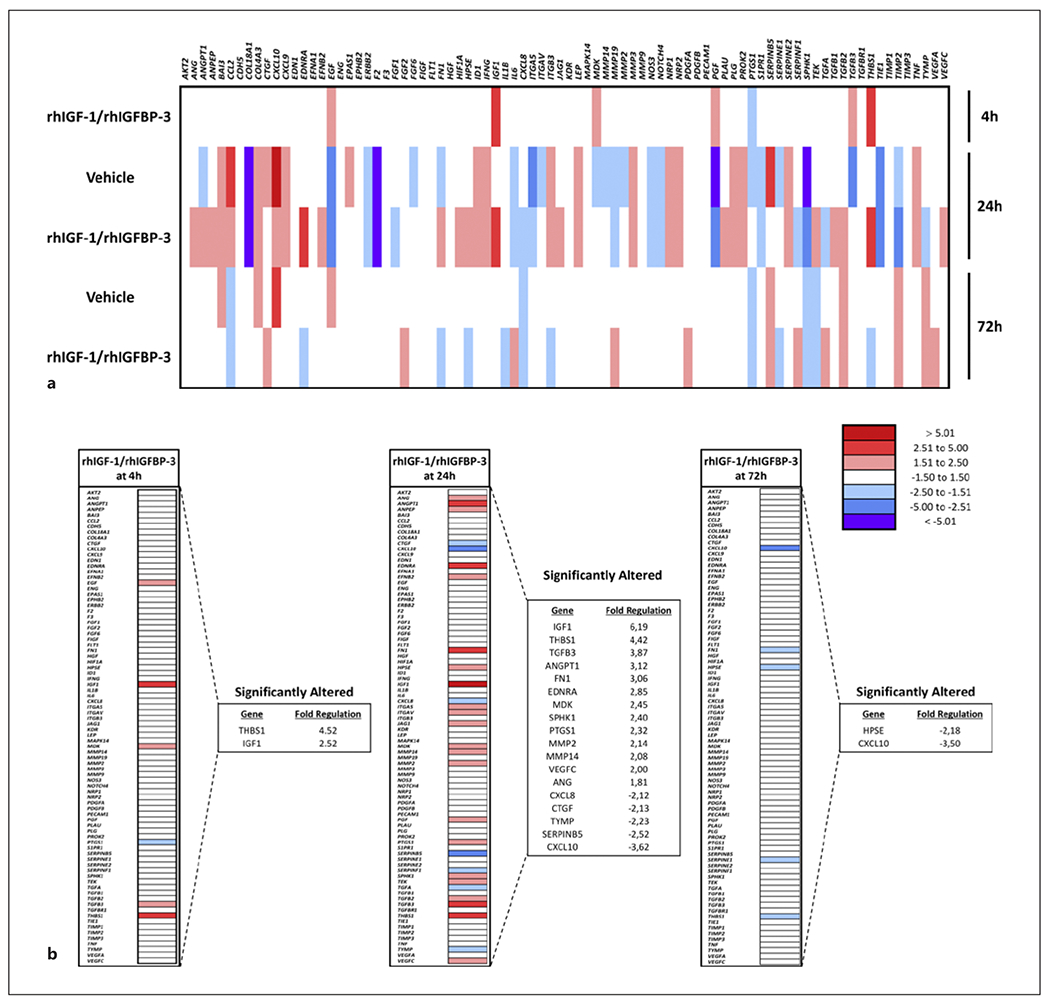
Angiogenesis array. The expression of 84 genes related to angiogenesis were evaluated using RT2 Profiler PCR arrays in choroid plexus tissue samples from preterm rabbit pups (E29) at 4, 24, and 72 h after exposure to 8 mg/kg/dose of rhIGF-1/rhIGFBP-3 or vehicle. Animals were given rhIGF-1/rhIGFBP-3 or vehicle from 3 h of age and every 12 h until 75 h of age (for a total of 1–7 administrations depending on the time of termination). Analysis was performed on pooled samples from pups terminated at 4 h (*n* = 6 in the vehicle group and *n* = 6 in the rhIGF-1/rhIGFBP-3 group) and 72 h (*n* = 7 in the vehicle group and *n* = 8 in the rhIGF-1/rhIGFBP-3 group) after first dosing or individual samples from pups terminated at 24 h (*n* = 6 in the vehicle group and *n* = 7 in the rhIGF-1/rhIGFBP-3 group) after first dosing. **a** Data were normalized to GAPDH, included in the RT2 Profiler PCR Arrays, and the fold change values were calculated by normalizing against those for vehicle-treated animals at 4 h. Data are visualized as fold change in a heat map. **b** Data were normalized to GAPDH, included in the RT2 Profiler PCR Arrays, and the fold change values were calculated by normalizing against the vehicle-treated group at the respective time points. Data are visualized as fold change in a heat map, and the most significantly altered genes (i.e., up- or downregulated more than 2-fold) at the respective time points are listed. rhIGF-1, recombinant human insulin-like growth factor 1; rhIGFBP-3, recombinant human insulin-like growth factor binding protein 3; GAPDH, glyceraldehyde-3-phosphate dehydrogenase.

Gene expression analysis of targets related to ECM and adhesion as well as angiogenesis was performed in the choroid plexus tissue following exposure to 8 mg/kg/dose of rhIGF-1/rhIGFBP-3 or vehicle from 3 h of age, and every 12 h until 75 h of age (for a total of 1–7 doses, depending on the time of termination; see online suppl. material for details). Analysis was initially performed using the RT2 Profiler Array of 84 genes related to ECM and adhesion (Fig. 4) or angiogenesis (Fig. 5) on pooled samples (from pups terminated at 4 and 72 h after first dosing) or individual samples (pups terminated at 24 h after first dosing). Results showed that the 8 mg/kg/dose of rhIGF-1/rhIGFBP-3 induced a general gene upregulation in both pathways and at all time points, as compared to the vehicle-exposed animals (Fig. 4, 5). Furthermore, in both profiler arrays, the most significant increase occurred at 24 h after the first dosing, whereas we detected the least change at 72 h. The target genes affected by rhIGF-1/rhIGFBP-3 were consistent between both profiler arrays and across the different time points.

Based on the results of the profiler array and earlier published findings, we selected the following targets for further specific qPCR analysis in individual samples: ANGPT1, IGF-1, FN1, COL1A1, VCAN, and THBS1. To a large extent, data from specific qPCR analysis confirmed the profiler array data, particularly showing that the most significant effect of rhIGF-1/rhIGFBP-3 was present at 24 h after the first dosing, that is, a significant upregulation of ANGPT1, IGF-1, and COL1A1 and a clear trend to upregulation of FN1 and VCAN as compared to vehicle-treated animals.

**Fig. 6.**
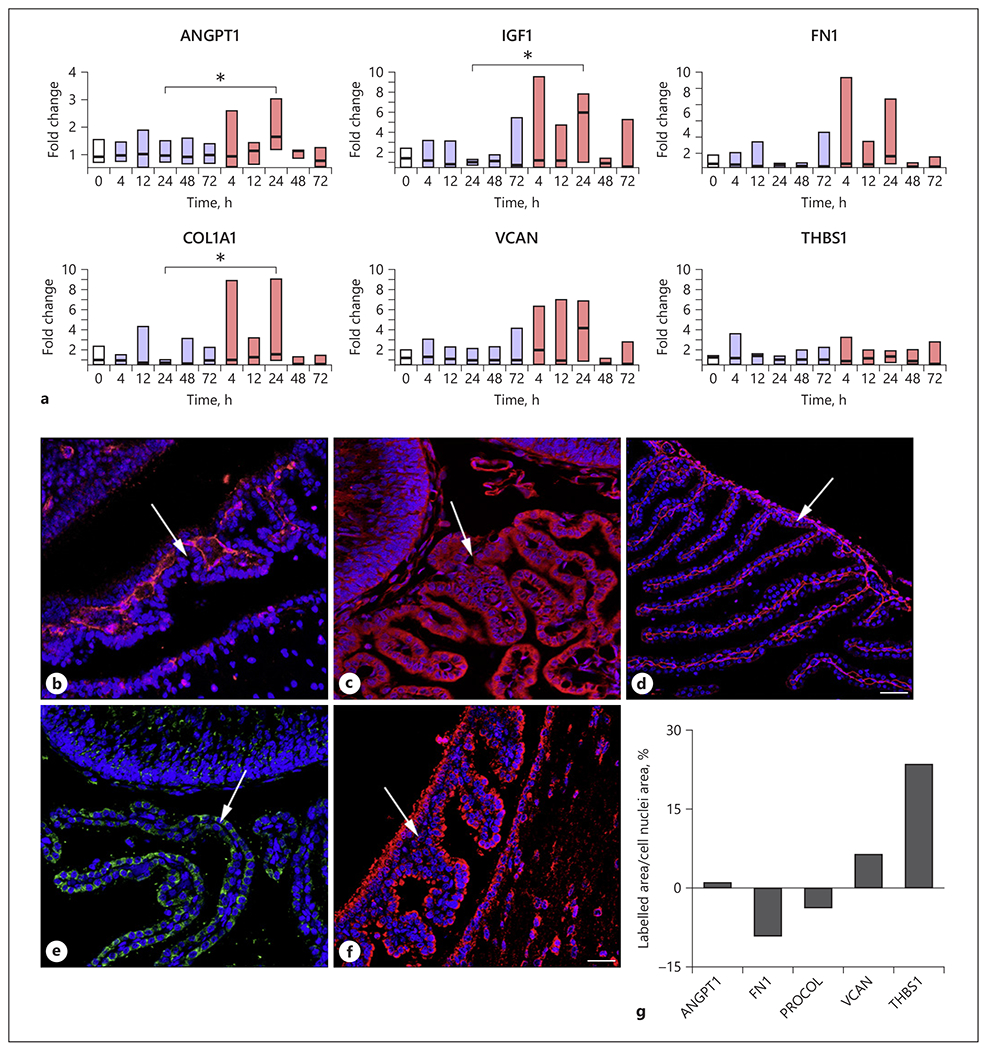
Detailed mRNA and protein analysis. **a** Preterm rabbit pups were exposed to 8 mg/kg/dose of rhIGF-1/rhIGFBP-3 (red bars) or vehicle (blue bars) from 3 h of age and every 12 h until 75 h of age (for a total of 1–7 administrations depending on the time of termination), and choroid plexus tissue samples were collected at 0, 4, 12, 24, 48, and 72 h after first dosing. qPCR analysis was performed on individual samples (*n* = 3–8) using the RT2 Primer Assay, and data were normalized to GAPDH. The fold change values were calculated by normalizing against values for vehicle-treated animals at 0 h (white bars), and data are visualized as fold change at the respective time points. Differences between rhIGF-1/rhIGFBP-3 versus vehicle-treated animals at the respective time points were analyzed using 1-way ANOVA with post hoc Sidak. *p < 0.05. **b–g** Preterm rabbit pups exposed to 8 mg/kg/dose of rhIGF-1/rhIGFBP-3 or vehicle were terminated at 48 h after first dosing, and brains were collected. Immunofluorescence labeling for confocal microscope analysis of ANGPT1 (**b**, red), FN1 (**c**, red), PROCOL (**d**, red), VCAN (**e**, green), and THBS1 (**f**, red) was performed as described in the Materials and Methods, and representative images from the choroid plexus/subfornical organ are shown. **g** A semiquantitative image analysis of respective protein targets in the choroid plexus, comparing rhIGF-1/rhIGFBP-3 versus vehicle-treated animals, was performed as described in the Materials and Methods. Scale bar in (**d**) represents 50 μm. Scale bar in (**f**) represents 50 μm and applies to **b**, **c**, **e**, **f**. Cell nuclei were counterstained with DAPI (blue). rhIGF-1, recombinant human insulin-like growth factor 1; rhIGFBP-3, recombinant human insulin-like growth factor binding protein 3; GAPDH, glyceraldehyde-3-phosphate dehydrogenase; ANGPT1, angiopoietin-1; FN1, fibronectin-1; PROCOL, procollagen-1; VCAN, versican; THBS1, thrombospondin-1; DAPI, 4′,6-diamidino-2-phenylindole.

To verify rhIGF-1/rhIGFBP-3-induced gene expression at the protein level, we performed immunofluorescent labeling for ANGPT1, FN1, PROCOL, VCAN, and THBS1. Regions of interest were analyzed and imaged using confocal microscopy. Because gene expression was significantly affected at 24 h after the first dosing, we evaluated a subsequent effect on protein levels at 48 h after the first dosing. All proteins exhibited positive staining and were present in the choroid plexus and subfornical organ in animals at 48 h after exposure to the 8 mg/kg/dose of rhIGF-1/rhIGFBP-3 (Fig. 6b–f). A semi-quantitative image analysis of the respective protein targets in the choroid plexus (see online suppl. material for details on image analysis), comparing the presence of immunofluorescence in vehicle-treated animals versus dosed animals 48 h after administration, indicated an increased protein level in some targets among the dosed group (Fig. 6g).

### Body Weight following s.c. Administration of rhIGF-1/rhIGFBP-3

We found no trend in body weight differences after exposure to rhIGF-1/rhIGFBP-3 (1–8 mg/kg/dose) as compared to vehicle-exposed animals (online suppl. Table 6).

## Discussion

Low postnatal serum levels of IGF-1 have been associated with an increased risk for neurodevelopmental impairment in preterm infants [1, 3, 4]. In this study, we characterized the IGF-1 system in the preterm rabbit pup, an animal model that mimics an essential aspect of IGF-1 physiology in preterm infants, namely an early postnatal decline in circulating levels of endogenous IGF-1 [20]. This characteristic enables evaluation of the effects of supplementary IGF-1 treatment in maintaining fetal levels following a premature transition to postnatal life. Here, we observed that administration of the protein complex rhIGF-1/rhIGFBP-3, in a dose sufficient to achieve in utero circulating levels of IGF-1, was linked to upregulated mRNA expression of factors involved in formation and regulation of cerebrovascular structure, morphogenesis, and maturation. Of note, we observed this upregulation in the choroid plexus, a structure we have previously described as an essential site for vessel rupture during IVH in the preterm rabbit pup [21, 22].

The inherent vulnerability to perturbations in cerebral blood flow resulting in IVH in the preterm infant is most pronounced during early postnatal life. This is also true for the preterm rabbit pup, in which the prevalence of spontaneous as well as hyperosmolarity-induced IVH decreases dramatically with increasing postnatal age [19, 23]. Consequently, a preventive treatment against IVH likely would require initiation prenatally or quite early after birth. Results of a recent clinical study support the impact of early administration. A non-significant trend toward decreased rates of severe IVH among preterm infants receiving a continuous i.v. infusion of rhIGF-1/rhIGFBP-3 from <24 h after birth was observed [16]. In the current study, we characterized the endogenous IGF-1 system and established the PK profile of exogenously administered rhIGF-1/rhIGFBP-3 during the immediate postnatal period in a preterm rabbit pup model. Similar to preterm infants [2], and as previously described for the preterm rabbit pup [20], we observed that circulating endogenous levels of IGF-1 in preterm rabbit pups decreased rapidly following cord ligation, reaching a 5-fold lower baseline level at approximately 72 h of age. Congruently, hepatic IGF-1 mRNA expression, representing the main source of circulating IGF-1 protein, increased only slightly with postnatal age, indicating that loss of placenta-maternal support is not compensated by endogenous production.

In repeated clinical studies, we have characterized the PK profile of rhIGF-1/rhIGFBP-3 in very preterm infants [16, 24–26]. The half-life of IGF-1 has been found to be short, approximately 2 h, necessitating continuous i.v. infusion as a route of administration. Data from the current study of s.c. administered rhIGF-1/rhIGFBP-3 in preterm rabbit pups indicated a similar PK profile and short half-life. The s.c. dose that led to circulating peak levels corresponding to those in utero was relatively high, 8 mg/kg, as compared to the human i.v. dose (250–400 μg/kg/24 h). Subsequently, a rapid elimination was observed, leading to trough levels considerably below target. In fact, an analysis just before subsequent dosing showed IGF-1 levels near the level of the vehicle-treated animals. As described, continuous i.v. administration is used in preterm infants, but this administration is difficult to achieve and maintain in the preterm rabbit pup and is a limitation on achieving stable circulatory concentrations. In the current study, based on ethical and practical considerations, we implemented twice daily s.c. dosing.

The biological effects of IGF-1 are mainly mediated by the IGF1R, a glycoprotein located on the cell surface, and which transduces the signal to a tail in the intracellular compartment of the signaling system leading to activation of the PI3K/Akt and Ras/ERK pathways [6, 7]. We observed that IGF1R was widely distributed within the naive newborn rabbit pup brain, with high densities in the choroid plexus, subfornical organ, meninges, periventricular zones, and major fiber tracts. We found no obvious difference between preterm and term pups on their respective first postnatal days. Following birth, however, IGF1R density decreased rapidly with increasing postnatal age, and by postnatal day 4, the choroid plexus and meninges were the only regions with relatively high levels of expression. These findings correspond well to those previously described in rodents, with a lingering expression in the choroid plexus and meninges in adult rats, in contrast to a wide-spread distribution during the embryonic period [27], suggesting that the transition to postnatal life has a stronger influence on cerebral IGF1R distribution than maturation per se.

When rhIGF-1/rhIGFBP-3-induced circulating IGF-1 levels reached their peak, extensive IGF-1 immunostaining was observed in brain regions with high IGF1R densities. Furthermore, double immunofluorescence labeling showed a clear coexistence of IGF-1 with IGF1R and was more intense in pups that had received rhIGF-1/rhIGFBP-3 as compared to vehicle-treated pups. Of note, the antibody used for labeling of IGF-1 does not discriminate between endogenous and exogenously administered IGF-1 (from rhIGF-1/rhIGFBP-3), precluding definitive conclusions concerning tissue distribution of administered IGF-1.

The fragility of the vasculature in the germinal matrix, a region prone to vessel rupture and bleeding in preterm IVH, is attributed to an augmented proliferative endothelial rate driven by increased expression of vascular endothelial growth factor (VEGF) and of angiopoietin-2 (ANGPT2). In support of this hypothesis, pharmacological suppression of VEGF and VEGF receptor-2 has been shown to decrease the rate of IVH in the preterm rabbit pup model (reviewed in [19]).

Studies in humans and rodents indicate that IGF-1 plays a key role in developmental angiogenesis, and deletion of the IGF1R gene leads to severe compromise of vascularization [28]. The process of angiogenesis covers the formation of new vessels as well as the stabilization and maturation of existing vessels. Studies in endothelial cell tubes and retinal neovessels have shown that VEGF and IGF-1 differ characteristically in their respective roles in vessel formation. Those in vitro studies showed that IGF-1 stabilizes nascent vessels whose formation was driven by VEGF and that ERK activation mediates the stabilizing effect of IGF-1 [29]. Here, we observed that systemic treatment with rhIGF-1/rhIGFBP-3 was associated with upregulation of mRNA in the choroid plexus of several genes described to play a role in promoting neovessel tubular organization, stabilization, and maturation, including ANGPT1, IGF-1, FN1, COL1A1, VCAN, and THBS1.

Following exposure to rhIGF-1/rhIGFBP-3, mRNA expression of the glycoprotein ANGPT1 was upregulated in the choroid plexus and the corresponding protein was observed in choroid plexus endothelium. Of interest, although ANGPT1 and ANGPT2 bind to the same endothelial Tie-2 receptor, they mediate opposing effects. While ANGPT1 has a restorative role in systemic vasculature and supports vascular maturation, in addition to its antiapoptotic effect, ANGPT2 has antagonistic effects and works in concert with VEGF, leading to a hyperproliferative state and reduced functional integrity [30].

The mRNA expression of IGF-1 was highly upregulated in the choroid plexus of pups exposed to rhIGF-1/rhIGFBP-3. A previous study has shown that IGF-1 mRNA, as well as protein secretion, is increased in endothelial cells following in vitro exposure to IGFBP-3. This effect is associated with phosphorylation of IGF1R and activation of the PI3K/Akt and Ras/ERK pathways [31]. Induction of endogenous IGF-1 expression has not previously been reported following exposure to rhIGF-1/rhIGFBP-3 in vivo.

THBS1 is a large oligomeric glycoprotein located in the ECM that was upregulated in the choroid plexus of pups exposed to rhIGF-1/rhIGFBP-3 in the current study. The thrombospondins participate in cell-cell interaction, differentiation, and proliferation and have antiangiogenic effects. THBS1 has been described as playing an important role in astrocyte-supported synaptogenesis and neuronal differentiation [32]. More recently, THBS1 has proved relevant for maintaining blood-brain barrier integrity following traumatic brain injury in adult rats [33]. In addition, THBS1, like integrin beta 8, induces activation of transforming growth factor β, a key component in blood vessel maturation and stabilization, supporting the assembly of the pericyte endothelial basement membrane matrix [34]. To the best of our knowledge, this study is the first animal study showing an upregulation of THBS1 in the choroid plexus.

Engineered deficiencies in structural components of the basal lamina including FN1, collagen, and laminin can induce vascular destabilization [35, 36]. FN1 and collagen 1 regulate the integrin-endothelial cell-cytoskeletal axis involved in precapillary cord formation and switching of sprouting morphogenesis to endothelial tubular maturation and stabilization. Furthermore, collagen and laminin production can be induced by the bioactive lipid sphingosine-1-phosphate, acting as a ligand regulating the integrin beta 8-transforming growth factor β-ECM-endothelium signaling axis in the brain [37, 38].

Severe cerebral IVH, with blood covering a major portion of the lateral ventricular space as determined by cranial ultrasound, remains one of the main challenges in clinical care of EPT infants. A recent nation-wide Swedish study comparing EPT infants born 2014–2016 to those born 2004–2007 showed that the prevalence of severe IVH in surviving infants at 1 year of age has remained unchanged at approximately 10% [9]. A recent phase 2 clinical study of rhIGF-1/rhIGFBP-3 treatment showed a trend toward decreased occurrence of severe IVH as compared to standard neonatal care [16]. However, the causal mechanisms involved in this treatment effect are not known. In the current study, we observed that s.c. administration of 8 mg/kg/dose of rhIGF-1/rhIGFBP-3 was associated with a significant presence of IGF-1 within important regions of the immature brain and induced an upregulation of angiogenesis- and ECM-related components. Altogether, this suggests that treatment with rhIGF-1/rhIGFBP-3 decreases vascular fragility and vulnerability to fluctuations in cerebral blood flow, reducing the risk for vessel rupture and induction of IVH.

## Conclusions

The preterm rabbit pup model is well-suited for evaluation of mechanisms involved in IGF-1-induced prevention of IVH. Administration of rhIGF-1/rhIGFBP-3 affects cerebrovascular maturation in critical brain regions, suggesting a role in prevention of preterm IVH.

## Supplementary Material

1
