# Supplementary material for: Insulin-Like Growth Factor 1 in the Preterm Rabbit Pup: Characterization of Cerebrovascular Maturation following Administration of Recombinant Human Insulin-Like Growth Factor 1/Insulin-Like Growth Factor 1-Binding Protein 3
Source: Dev Neurosci. Author manuscript; Available in PMC 2021 Nov 26. (PMC8623584; doi:10.1159/000516665)
Supplement: 1 [file NIHMS1756969-supplement-1.pdf]

Supplementary Material

Insulin-like growth factor 1 (IGF-1) in the preterm rabbit pup: Characterization of cerebro-vascular maturation following administration of recombinant human IGF-1/IGF binding protein 3

Magnus Gram[a], Claes Ekström[a], Bo Holmqvist[b], Galen Carey[c], Xiaoyang Wang[d], Suvi Vallius Kvist[a], William Hellström[e], Niklas Ortenlöf[a], Alex Adusei Agyemang[a], Lois E. H. Smith[f], Ann Hellström[g], Alexandra Mangili[h], Norman Barton[c], David Ley[a]

[a] Lund University, Department of Clinical Sciences Lund, Pediatrics, Lund, Sweden

[b] ImaGene-iT AB, Lund, Sweden

[c] Takeda Pharmaceuticals, Boston, MA, USA

[d] Institute of Neuroscience and Physiology, Sahlgrenska Academy, University of Gothenburg, Gothenburg, Sweden

[e] Department of Pediatrics, Institute of Clinical Sciences, Sahlgrenska Academy, University of Gothenburg, Gothenburg, Sweden

[f] Department of Ophthalmology, Boston Children's Hospital, Harvard Medical School, Boston, MA, USA

[g] Department of Clinical Neuroscience, Sahlgrenska Academy, University of Gothenburg, Gothenburg, Sweden

[h] Global Clinical Development, Rare Metabolic Diseases, Shire, a Takeda company, Zurich, Switzerland

Supplementary Materials and Methods

Animals

The animal protocols were approved by the Swedish Animal Ethics Committee in Lund, Sweden. Reporting is in compliance with the ARRIVE guidelines. We used the well-established preterm rabbit pup model in accordance with previous description[1]. A half-breed between New Zealand White and Lop was used (Christer Månsson, Löberöd, Sweden). Briefly, the experiments were performed on a total of 306 rabbit pups from 55 litters (168 females and 138 males, for details see Supplementary Table 1) delivered via cesarean section (c.s.) after the does were anesthetized with intravenous propofol (5 mg/kg, Primen Pharmaceuticals Oy, Helsinki, Finland) on day 29 (term 31-32 days). After delivery, the pups were handled and nursed by animal laboratory staff. The pups were dried and placed in an infant incubator set to 30°C and 60% humidity. At approx. 1-2 hours of age, the pups were weighed, marked, and hand-fed with bovine colostrum (100 ml/kg/day, Biodane Pharma, Gesten, Denmark) using a 3.5 French feeding tube (Vygon, Ecouven, France) and fed every 12 hours. The administered amount of colostrum was increased every 24 hours by 30 ml/kg/day. Pups were cleaned once or twice a day, as determined necessary to maintain hygiene. In one of the sub-sections of these studies (see experimental setup, Stage 1, below for more details), rabbit pups were delivered vaginally at term (31-32 days). These pups were cared for and fed by the doe throughout the study.

Experimental setup

Characterization of endogenous serum IGF-1, liver IGF-1 mRNA, and brain IGF-1 and IGF1R

Stage 1: Immediately following c.s., the preterm rabbit pups were marked and randomized to one of two groups. Group 1: Pups were weighed and head circumference (HC) measured. Pups were terminated at < 5 minutes of age and blood, liver, and ear samples were collected from all pups and processed as described below. Group 2: Pups were weighed and HC measured. At approx. 24 hours of age, following sedation with intraperitoneal ketamine administration (35 mg/kg, Intervet International B.V, Boxmeer, The Netherlands) and isoflurane inhalation (1000 mg/g, Virbac Carros, France), a blood sample was obtained through heart puncture (processed as described below), and the pups were transcardially perfused with 0.9% saline (containing heparin, 100 IU/ml) followed by biopsy sampling of the liver and ear. Samples were snap frozen on dry ice and processed as described below. Following saline perfusion, pups were perfused with freshly prepared 4% paraformaldehyde (PFA, in 0.1 M phosphate buffer, pH 7.4) and the brains dissected out from the skull, immersed in 4% PFA, and processed as described below. In addition to the preterm groups (groups 1 and 2), a third term rabbit pup group was included in this section. Group 3: Term pups (31-32 days) were vaginally delivered and within the first 5-7 hours of age, following sedation as described above, the pups were weighed, HC measured, and a blood sample obtained through heart puncture (processed as described below). The

pups were transcardially perfused with 0.9% saline (containing heparin, 100 IU/ml) followed by biopsy sampling of the liver and ear. Samples where snap frozen on dry ice and processed as described below. Following saline perfusion, pups were perfused with freshly prepared 4% PFA, and the brains were dissected out from the skull, immersed in 4% PFA, and processed as described below.

Stage 2: Immediately following c.s., the preterm rabbit pups were marked and randomized, with regards to time-point of termination, to one (1) out of seven (7) groups. Group 1: Pups were weighed and HC measured. Pups were terminated at $<$ 5 minutes of age, and blood, liver, and ear samples were collected from all pups and processed as described below. Groups 2-7: Pups were weighed and HC measured. At approx. 2, 4, 8, 24, 48, and 72 hours of age, respectively, pups were terminated and a blood sample (processed as described below) and liver and ear biopsy samples (snap frozen on dry ice and processed as described below) were collected.

<u>Characterization of serum IGF-1, liver IGF-1, and brain IGF-1 and IGF1R following exposure to rhIGF-1/rhIGFBP-3</u>

Stage 3: Immediately following c.s., the preterm rabbit pups were marked and randomized with regard to administration of rhIGF-1/rhIGFBP-3 (supplied, formulated, and prepared in vehicle solution as described by Takeda Pharmaceutical Company Ltd, Boston, MA, USA) or saline vehicle (NaCl Braun, 9 mg/ml sterile isotonic saline, B Braun, Danderyd, Sweden) and time-point of termination, to one (1) out of fourteen (14) groups. Group 1: Pups were weighed and HC measured. Pups were terminated at $<$ 5 minutes of age, and blood, liver, and ear samples were collected from all pups and processed as described below. Groups 2-14: Pups were weighed and HC measured. At approx. 72 hours of age, the pups received a 0.1 ml subcutaneous (s.c.) dose of 1.0-8.0 mg/kg rhIGF-1/rhIGFBP-3 or vehicle (9 mg/ml NaCl, B Braun) and were followed up to 48 hours post-administration. At approx. 0 (72), 0.5 (72.5), 2.0 (74), 6.0 (78), 8.0 (80), 24 (96), and 48 (120) hours post rhIGF-1/rhIGFBP-3 or vehicle administration (corresponding postnatal age within brackets), the pups were terminated and a blood sample (processed as described below) and liver and ear biopsy samples (snap frozen on dry ice and processed as described below) were collected. A subset of animals was, following sedation as described above, terminated at approx. 0.5 and 24 hours post rhIGF-1/rhIGFBP-3 or vehicle administration. Following perfusion with 0.9% saline followed by freshly prepared 4% PFA, the brains were dissected out from the skull, immersed in 4% PFA, and processed as described below.

Stage 4: Immediately following c.s., the preterm rabbit pups were marked and randomized with regard to administration of rhIGF-1/rhIGFBP-3 or vehicle (9 mg/ml NaCl, B Braun) and time-point of termination to one (1) out of fourteen (14) groups. Group 1: Pups were weighed and HC measured. Pups were terminated at $<$ 5 minutes of age and blood and liver and ear samples were collected from all pups and processed as described below. Groups 2-14: Pups were weighed and HC measured. At

approx. 3 hours of age, the pups received a 0.1 ml s.c. dose of 8.0 mg/kg rhIGF-1/rhIGFBP-3 or vehicle (9 mg/ml NaCl, B Braun) that was repeated every 12 hours up to approx. 72 hours post administration (one (1) to seven (7) administrations of rhIGF-1/rhIGFBP-3 or vehicle in total). At approx. 0 (0), 4 (7), 12 (15), 24 (27), 48 (51), and 72 (75) hours post rhIGF-1/rhIGFBP-3 or vehicle administration (corresponding postnatal age within brackets), the pups were terminated and samples collected for choroid plexus gene expression analysis according to the following procedure: At the respective time-points, the pups were sedated as described above, followed by opening of the chest and apical cardiac puncture perfusion with 50 ml of 0.1 M phosphate buffered saline (PBS, pH 7.4) for 30 seconds (until visible bleaching of the tissue was observed). After completion of perfusion, the pups were terminated by cervical dislocation and the brains dissected out from the skull and placed in an ice-cold petri dish containing 6 ml Krebs-Ringer HEPES solution (Alfa Aesar, Kandel, Germany). By sharp dissection, the hemispheres were divided and the choroid plexus removed from the ventricular cavity by blunt dissection with a pincette. The choroid plexus tissue biopsy, as well as a liver and ear biopsy samples collected at termination, were snap frozen on dry ice and processed as described below. From all animals, a blood sample was collected at the respective termination time-points and processed as described below. At approx. 4 (7), 24 (27), 48 (51), and 72 (75) hours post rhIGF-1/rhIGFBP-3 or vehicle administration (corresponding postnatal age within brackets), the pups were terminated and samples collected for histochemistry and immunohistochemistry analysis according to the following procedure: Following sedation as described above, the pups were perfused with 0.9% saline, followed by transcardial perfusion with freshly prepared 4% PFA, and the brains were dissected out from the skull, immersed in 4% PFA, and processed as described below.

Tissue collection and processing for histology and immunolabeling

Following fixation, the brain tissue was dehydrated in a graded ethanol series (70-99.99%), followed by xylene (100%), and finally immersed in paraffin and embedded in paraffin blocks. Coronal and sagittal brain sections (4 or 6 µm) were prepared on a rotating microtome (Microm HM 360, Microm International GmbH, Walldorf, Germany), and sections were collected on microscope slides (SuperFrost Plus, Thermo Scientific/Gerhard Menzel B.V. & Co., Braunschweig, Germany).

Immunohistochemistry

The specific labeling of the primary antibodies against IGF1R and IGF-1 was confirmed by the distribution of labeling in rabbit brain tissue, the distribution of labeling in rabbit (not shown) and human placenta tissue (i.e. positive control tissue), and the lack of labeling when excluding the primary antibody from the protocol (i.e. negative control) (Supplementary Fig. 2). The primary antibody used against IGF1R, a polyclonal goat anti-IGF1R (AF-305-NA #639h, R&D System, McKinley Place, MN, USA), provided strong labeling with low background (using both chromogen immunohistochemistry and

immunofluorescence). The primary antibody used against IGF-1, a monoclonal mouse anti-IGF-1 (AM33345PU-S, Origene, Herford, Germany), provided a strong but less distinct labeling. For chromogen visualization of IGF-1 immunoreactive sites, nickel ion intensification of the immunoreaction was required.

For IGF1R and IGF-1 immunolabeling, sections were de-paraffinized and rehydrated, starting in xylene followed by a graded ethanol series ended in 0.1 M PBS, pH 7.4. Sections (4-6 μm) were then quenched for endogenous peroxidase activity, in hydrogen peroxidase (0.1%) for 10 minutes, followed by incubation in PBS containing 0.05% Triton X-100 (TX) and 1% bovine serum albumin (BSA) for 30 minutes at room temperature. Sections were then incubated with IGF1R or IGF-1 primary antibody (2 μg/ml diluted in PBS-TX-BSA) for 16 hours at 4°C. Following rinses in PBS-TX, sections were incubated with secondary horseradish peroxidase (HRP) conjugated antibodies (donkey anti-goat IgG, diluted 1:750 in PBS-TX-BSA, Jackson IR, West Grove, PA, USA; or with goat anti-mouse IgG (K4001), DAKO Envision, Agilent, Santa Clara, CA, USA, diluted 1:1 in PBS-TX-BSA), for 30 minutes at room temperature. All antibody incubations were performed in a moisture chamber. For the chromogen visualization of the IGF1R immunoreactive sites, the HRP conjugation was reacted in a PBS solution containing diaminobenzidine (0.5 mg/ml) and hydrogen peroxidase (0.1%) for 10 minutes at room temperature. IGF1R immunolabeled sections were counterstained with hematoxylin, whereas IGF-1 labeled sites were not counterstained (illustrated with differential interference contrast in Supplementary Fig. 2). Sections were then rinsed in PBS, dehydrated (graded ethanol ending with 100% xylene), mounted (Pertex, Histolab, Gothenburg, Sweden), and cover-slipped.

The chromogen labeled coronal and sagittal sections were analyzed by visual inspection of microscope slides in a bright-field microscope (Olympus IX73, Shinjuku, Tokyo, Japan). Whole sections were scanned (Hamamatsu, NanozoomerS60, Hamamatsu, Japan), and digital image processing was performed to visualize the IGF1R neuroanatomical distribution and differences in receptor densities in the brain. An ImageJ macro was developed, and the threshold for detection of IGF1R immunoreactivity was set from the control slides (no primary antibody incubation). For quantitative analysis and comparison between animal groups, the IGF1R density in digital images of the IGF1R immunohistochemistry labeling in whole coronal sections (4-7 sections per animal) was analyzed. Artifacts (reaction precipitates and damaged tissue) were extracted from the images. Using the set threshold value, IGF1R densities per tissue area were measured, and the percentage of labeling was compared between the groups (not shown). Color coded images were made for illustrations of the differentiated receptor densities in the brain, represented as high (red), medium (green), low (blue), and no staining (black) (see Fig. 2A-B, Supplementary Fig. 2).

Immunofluorescence

Immunofluorescence labeling was used to elucidate the detailed distribution of IGF1R and for evaluation of the relation between IGF1R and IGF-1, endogenous and/or from administered rhIGF-1/rhIGFBP-3, as well as IGF-1 and the endothelial cell marker CD31. Single (IGF1R) or double labeling (IGF1R vs. IGF-1 or IGF-1 vs. CD31) was performed by using the IGF1R and IGF-1 antibodies as described above and a goat CD31 polyclonal IgG antibody (2 µg/ml diluted in PBS-TX-BSA, sc-1505, Santa Cruz Biotech, Dallas, TX, USA).

Immunofluorescence labeling was also used for detection and visualization of proteins selected from the gene expression analysis following exposure to rhIGF-1/rhIGFBP-3. The following primary antibodies were used: angiopoietin-1 (ANGPT1, goat polyclonal IgG, 2 µg/ml diluted in PBS-TX-BSA, PA5-46968, Invitrogen, Carlsbad, CA, USA), fibronectin-1 (FN1, mouse monoclonal IgG, 3 µg/ml diluted in PBS-TX-BSA, NBP2-22113SS, Novus Biological, Centennial, CO, USA), procollagen-1 (PROCOL, rat monoclonal IgG, ab64409, diluted 1:200 in PBS-TX-BSA, Abcam, Cambridge, UK), versican (VCAN, mouse monoclonal IgM, MABT161, 3 µg/ml diluted in PBS-TX-BSA, Millipore, Temecula, CA, USA), and thrombospondin-1 (THBS1, mouse monoclonal IgG, 5 µg/ml diluted in PBS-TX-BSA, MA5-13398, Thermo Fisher, Waltham, MA, USA).

For the immunofluorescence labeling, de-paraffinized and rehydrated sections were rinsed in PBS containing TX (0.05%), followed by blocking in PBS-TX-BSA for 30 minutes at room temperature. Sections were then incubated with the primary antibodies for 16 hours at 4°C. Following rinses in PBS-TX, sections were incubated for 45 minutes at room temperature with fluorophore conjugated secondary antibodies against the species specific primary antibody IgG (IGF1R, IGF-1, CD31, ANGPT1, FN1, PROCOL, and THBS1) or IgM (VCAN). The secondary antibodies were conjugated as follows: anti-goat AF488, anti-goat AF647, anti-mouse RodamineRX, AF568 or AF647 and anti-rat RodamineRX (all made in donkey, from Jackson ImmunoResearch, West Grove, PA, USA, diluted 1:200 in PBS-TX-BSA). Double labeling of IGF1R and IGF-1 and of IGF-1 and CD31 was performed as antibody cocktails, i.e. incubation with a mixture of primary antibodies and secondary antibodies, respectively. Sections were then rinsed in PBS-TX and incubated in 4′,6-diamidino-2-phenylindole (DAPI) for 15 minutes at room temperature, followed by rinsing in PBS and mounting in anti-fade solution (Abcam). In every run, primary antibody specificity was tested by omitting the primary antibody incubation from the protocol on adjacent sections to those incubated with primary antibodies (i.e. negative control, not shown). These control sections were also used to set the background threshold level for fluorescence detection, i.e. only fluorescence signal levels above the threshold were recorded. Confocal laser scanning analysis and imaging of the fluorescence labeling were performed using a Zeiss LSM 800 microscope (Zeiss, Germany) with x20 dry or x40 oil immersion lenses. Sequential scanning was performed with an optimized pinhole (airy unit) for each wavelength/fluorophore. For the double labeling analysis, the

pinholes were optimized for all channels to ensure detection and visualization of the individual labeling at the same focal plane. For quantitation of ANGPT1, FN1, PROCOL, VCAN, and THBS1, confocal images were grabbed of the region of interest, i.e. choroid plexus (4-8 images per animal) and exported to TIFF format. The area of interest was specifically selected and annotated manually. An ImageJ macro was developed to extract the immunofluorescence labeled targets, providing the total analyzed area and the DAPI labeled cell nuclei area. The immunofluorescence labeled area was measured and calculated per cell nuclei area (percentage), which was used for comparison between rhIGF-1/rhIGFBP-3 and vehicle treated animals.

RNA isolation, gene array, and real-time PCR

Total RNA was isolated from liver and choroid plexus tissue using RNeasy Mini Kit with on-column DNase digestion supplied by Qiagen (Hilden, Germany). The OD ratio (optical density at 260 nm/280 nm) of RNA was always higher than 2.0. Reverse transcription was performed according to the manufacturer on 0.5-1.0 μg total RNA using RT2 First Strand Kit (Qiagen, used for RT2 Profiler PCR Array analysis as described below) and iScript cDNA Synthesis Kit (Bio-Rad, Hercules, CA, USA). The expression of 84 genes related to extracellular matrix (ECM) and adhesion molecules (RT2 Profiler PCR Array, Rabbit Extracellular Matrix and Adhesion Molecules, Cat. No. 330231 PANZ-013Z, Qiagen) and angiogenesis (RT2 Profiler PCR Array, Rabbit Angiogenesis, Cat. No. 330231 PANZ-024Z, Qiagen) were evaluated using pooled (4 and 72 hours) or individual (24 hours) choroid plexus tissue samples from the respective groups. The RT2 SYBR Green qPCR Mastermix (Qiagen) was used to quantify mRNA expression. Data were normalized to glyceraldehyde-3-phosphate dehydrogenase (GAPDH, included in the RT2 Profiler Arrays, Qiagen), and the fold change values were calculated by normalizing against the representative vehicle treated animals (see respective Fig. for details). Data were visualized as fold change in a heat map, and the most significantly altered genes (i.e. up- or downregulated more than 2 fold) at the respective time-points are listed.

Based on the outcome of the profiler arrays, a number of significantly altered genes were further evaluated in specific qPCR analysis using RT2 Primer assay (all from Qiagen). The selected target genes were collagen, type I, alpha 1 (COL1A1, Cat No. PPN09687A), IGF-1 (Cat. No. PPN00061A), FN1 (Cat. No. PPN01149A), VCAN (Cat no. PPN05138A), THBS1 (Cat. No. PPN03605A), and ANGPT1 (Cat. No. PPN14543A). Expression was analyzed using the iTaq Universal SYBR Green Supermix (Bio-Rad), data were normalized against GAPDH (Qiagen, Cat. No. PPN00377A), and the fold change values were calculated by normalizing against representative control samples from vehicle treated animals (see respective Fig. for details). Data are visualized as fold change at the respective time-points. Amplification was performed as described by the manufacturer (Qiagen, RT2 SYBR Green qPCR

Mastermix and Bio-Rad, iTaq Universal SYBR Green Supermix) for 40 cycles (both assay formats) in an CFX Connect (Bio-Rad). Data were analyzed using Bio-Rad CFX Maestro 1.1 (Bio-Rad).

Sex determination

Rabbit sex was determined by confirmation of the presence of the sex determining region Y gene (SRY, gene ID: 100328958) sequence in the rabbit genome using PCR and visualization by gel electrophoresis. DNA was extracted using the DNeasy Blood and Tissue Kit (Qiagen) according to the manufacturer's instructions. One (1) μl of DNA was used in respective PCR reaction (30 cycles at 57°C) with the following primers: Sense: TGCAATACAGGAGGAACACG, Antisense: AGCAAACTGTCGCTCTTCTG. The presence of a band at approx. 299 bp was determined as male, and correspondingly no visual band was determined as female.

Supplementary References

1. Sveinsdóttir S, Cinthio M, Ley D. High-frequency ultrasound in the evaluation of cerebral intraventricular haemorrhage in preterm rabbit pups. Ultrasound Med Biol. 2012 Mar;38(3):423-31.

Supplementary Figure Legends

Supplementary Fig. 1. Schematic summary of the experimental setup. The details of the experiments are described in the Materials and Methods; below is a brief summary of the details and a description of the abbreviations and symbols included in the figure. Following birth, preterm (cesarean section, C.S.) or term (vaginal delivery, V.D.) rabbit pups were handled and nursed by either the animal laboratory staff (preterm pups) or the doe (term). Pups were randomized to the respective experimental groups, treatment regimens, and termination time-points. In experimental outline stages 3 and 4, rhIGF-1/rhIGFBP-3 or the corresponding vehicle was s.c. administered once (1) or repeatedly at a dose of 1.0-8.0 mg/kg/dose (at a fixed volume of 0.1 ml/animal/dose). Dosing of preterm pups was started either at approx. 3 hours of age (stage 4) and repeated every 12 hours up to approx. 75 hours of age (one (1) to seven (7) administrations of rhIGF-1/rhIGFBP-3 or vehicle in total), or starting at approx. 72 hours of age (stage 3, one (1) administration of rhIGF-1/IGFBP-3 or vehicle in total). Pups were followed for 0-120 hours, terminated at the time-points indicated in Figures 1-6, and one or several of the following samples were collected and analyzed as described in the Materials and Methods: blood, used for determination of serum IGF-1 levels; liver biopsy, used for IGF-1 gene expression analysis; brain, used for IGF-1, IGF1R, and cerebro-vascular immunolabeling; choroid plexus, used for ECM- and angiogenesis-related gene expression analysis; and an ear biopsy, used for sex determination. Red arrows = indicate time-point of administration of rhIGF-1/rhIGFBP-3 or vehicle; black arrows = indicate time-point for termination and/or sampling; C.S. = cesarean section; V.D. = vaginal delivery; GA = gestational age; E = embryonic day; P = postnatal day; brackets = indicate that it covers several time-points for termination and/or sampling within the marked period; see Figures 1-6 for details.

Supplementary Fig. 2. IGF1R and IGF-1 immunoreactivity specificity. The specific labeling pattern of primary antibodies against IGF1R (brown, **A** and **B**, left) and IGF-1 (blue, **C** and **D**, left) was confirmed by the localization of labeling in rabbit brain tissue (**A** and **C**) and human placenta tissue (**B** and **D**), i.e. positive control tissue. The specificity of primary antibodies used was further confirmed by the lack of labeling when excluding primary antibody against IGF1R (**A** and **B**, right) and IGF-1 (**C** and **D**, right). **E.** Digital image processing of IGF1R chromogen labeled (brown, left) whole sections was performed to elucidate the IGF1R neuroanatomical distribution and differences in receptor densities in the brain, and color coded images (right) were made for illustrations of the differentiated receptor densities in the brain, represented as high (red), medium (green), low (blue), and no staining (black).

Supplementary Tables

Supplementary Table 1. Description of animals

Study	No. of pregnant does	No. of pups delivered	No. of pups included in studies (%)	No. of female pups (%)	No. of male pups (%)
Stage 1	6	22	22 (100)	18 (82)	4 (18)
Stage 2	8	72	61 (85)	34 (56)	27 (44)
Stage 3	27	196	130 (66)	60 (46)	70 (54)
Stage 4	14	115	93 (81)	56 (60)	37 (40)
**Total**	**55**	**405**	**306 (76)**	**168 (55)**	**138 (45)**

Supplementary Table 2. Body weight and head circumference

Timepoint	Body weight preterm pups (g)[1]	Body weight term pups (g)[1, 3]	Head circumference preterm pups (mm)[1, 2]	Head circumference term pups (mm)[1, 3]
T=0h (E29 + 0h)	40 (24-58, n=298)***[2]	-	16 (13-21, n=277)***[2]	-
T=24h (E29 + 24h)	42 (25-59, n=217)***[2]	-	-	-
T=48h (E29 + 48h)	42 (25-58, n=178)***[2]	-	-	-
T=72h (E29 + 72h)	43 (25-61, n=154)***[2]	-	-	-
T=96h (E29 + 96h)	42 (28-58, n=48)***[2]	-	-	-
T=120h (E29 + 120h)	41 (31-47, n=19)***[2]	-	-	-
T=Term (P0 + 0h)	-	54 (49-56, n=6)	-	20 (19-20, n=6)

[1]Data are presented as median (range, n).

[2]Differences between term pups (P0 + 0h) vs. preterm pups at respective time-points were analyzed using one-way ANOVA with post hoc Bonferroni.

***$P<0.001$.

[3]Weight and head circumference obtained at approx. 5-7 hours after term vaginal delivery. All animals from same litter.

n = Number of pups, h = hours, g = gram, mm = millimeter.

Supplementary Table 3. Serum IGF-1 protein and liver IGF-1 mRNA

**Timepoint**[1]	**Serum IGF-1 (ng/ml)**[2]	**Liver IGF-1 mRNA**[5]
T=0h (E29 + 0h)	149.8 (93.1-220.4, n=27)***[4]	1.0 (0.4-1.8, n=12)***[4]
T=2h (E29 + 2h)	134.8 (106.2-162.4, n=9)***[4]	-
T=4h (E29 + 4h)	123.0 (81.3-135.5, n=9)**[3], *[4]	-
T=8h (E29 + 8h)	116.0 (100.9-154.4, n=9)*[3], *[4]	-
T=24h (E30, E29 + 24h)	68.9 (38.3-111.7, n=19)***[3], **[4]	2.4 (0.7-7.7, n=10)*[3]
T=48h (E31, E29 + 48h)	45.1 (38.9-70.4, n=8)***[3], **[4]	-
T=72[6] (P0, E29 + 72h)	31.5 (19.8-69.0, n=37)***[3], ***[4]	1.3 (0.2-7.7, n=28)
T=96h (P1, E29 + 96h)	24.8 (22.3-29.0, n=3)***[3], ***[4]	0.4 (0.3-2.3, n=4)*[4]
T=120h (P2, E29 + 120h)	48.1 (23.2-76.0, n=6)***[3], ***[4]	3.9 (2.4-5.2, n=4)***[3]
T=Term (P0 + 0h)	101.8 (81.0-111.2, n=6)	2.1 (1.6-3.9, n=6)

[1]Sample obtained at termination, i.e., one serum IGF-1/liver mRNA value at one timepoint was obtained per animal.

[2]Endogenous serum IGF-1 without exogenous administration of rhIGF-1/rhIGFBP-3. Data are presented as median (range, n).

[3]Differences between preterm pups at birth (T=0h, E29 + 0h) vs. preterm pups at respective time-points were analyzed using one-way ANOVA with post hoc Bonferroni. *$P<0.05$, **$P<0.01$, ***$P<0.001$.

[4]Differences between term pups (P0 + 0h) vs. preterm pups at respective time-points were analyzed using one-way ANOVA with post hoc Bonferroni. *$P<0.05$, **$P<0.01$, ***$P<0.001$.

[5]Data are presented as median fold change (range, n) vs. expression levels at T=0h (E29 + 0h). Data were normalized to ACTB.

[6]Samples collected at approx. 72-80 hours of age.

n = Number of pups, h = hours, ng = nanogram, ml = milliliter.

Supplementary Table 4. Serum IGF-1 following exposure to rhIGF-1/rhIGFBP-3[1]

Timepoint[2]	Vehicle[3]	1.0 mg/kg rhIGF-1/rhIGFBP-3[3]	2.0 mg/kg rhIGF-1/rhIGFBP-3[3]	4.0 mg/kg rhIGF-1/rhIGFBP-3[3]	8.0 mg/kg rhIGF-1/rhIGFBP-3[3]
T=0h (E29 + 72h)	42.5 (24.4-117.9, n=10)	-	-	-	-
T=0.5h (E29 + 72.5h)	-	-	71.9 (60.5-85.0, n=5)	124.4**[4] (75.5-141.5, n=3)	153.4***[4] (129.8-158.3, n=4)
T=1h (E29 + 73h)	-	47.8 (27.6-59.0, n=5)	79.00**[4] (67.2-102.6, n=8)	89.8*[4] (88.6-125.8, n=3)	177.86***[4] (130.4-221.6, n=3)
T=2h (E29 + 74h)	-	-	71.1*[4] (58.4-142.6, n=8)	-	-
T=4h (E29 + 76h)	-	43.1 (36.5-46.2, n=5)	-	102.6**[4] (102.5-104.6, n=3)	181.5***[4] (180.0-263.7, n=3)
T=6h (E29 + 78h)	-	-	56.6 (44.4-61.9, n=5)	-	-
T=8h (E29 + 80h)	-	-	45.7 (41.9-55.1, n=5)	-	-
T=24h (E29 + 96h[5])	-	-	29.4 (23.1-58.2, n=7)	60.6 (49.4-77.2, n=4)	70.5 (42.7-84.1, n=5)
T=48h (E29 + 120h[4])	-	-	23.6 (22.1-32.3, n=3)	46.0 (25.5-53.2, n=3)	53.4 (37.4-73.2, n=4)

[1]rhIGF-1/rhIGFBP-3 was administered at approx. 72 hours of age.

[2]Sample was obtained at termination, i.e. one serum IGF-1 value at one timepoint was obtained per animal. Timepoint is presented as time after rhIGF-1/rhIGFBP-3 administration (corresponding age of pup at sampling/termination).

[3]Data is presented as median (range, n).

[4]Differences between vehicle treated pups at T=0h (E29 + 72h) vs. rhIGF-1/rhIGFBP-3 treated pups at respective time-points were analyzed using one-way ANOVA with post hoc Bonferroni. *$P<0.05$, **$P<0.01$, ***$P<0.001$.

[5]Samples collected at approx. 96-120 hours of age.

n = Number of pups, h = hours, mg = milligram, kg = kilogram.

Supplementary Table 5. Liver IGF-1 mRNA expression following exposure to rhIGF-1/rhIGFBP-3[1]

Timepoint[2]	Vehicle[3]	1.0 mg/kg rhIGF-1/rhIGFBP-3[3]	2.0 mg/kg rhIGF-1/rhIGFBP-3[3]	4.0 mg/kg rhIGF-1/rhIGFBP-3[3]	8.0 mg/kg rhIGF-1/rhIGFBP-3[3]
T=0h (E29 + 72h)	1.5 (0.2-7.7, n=7)	-	-	-	-
T=0.5h (E29 + 72.5h)	1.3 (0.5-1.3, n=3)	-	2.8*[4] (1.5-3.7, n=5)	4.2 (0.7-10.2, n=3)	2.9*[4] (1.9-3.7, n=4)
T=1h (E29 + 73h)	1.4 (0.7-2.1, n=6)	1.7 (1.1-2.1, n=5)	1.3 (0.3-4.2, n=10)	1.6 (0.9-2.1, n=3)	2.6 (0.9-3.3, n=3)
T=2h (E29 + 74h)	1.1 (0.5-1.2, n=3)	-	1.3 (0.7-3.5, n=5)	-	-
T=4h (E29 + 76h)	2.9 (1.8-6.7, n=3)	1.7 (1.3-5.3, n=5)	-	1.4 (1.3-2.1, n=3)	2.7 (1.1-3.7, n=3)
T=6h (E29 + 78h)	1.1 (0.4-1.5, n=3)	-	2.1 (0.6-2.8, n=5)	-	-
T=8h (E29 + 80h)	1.0 (0.8-2.1, n=3)	-	1.6 (0.4-3.8, n=5)	-	-
T=24h (E29 + 96h[5])	1.4 (0.3-5.2, n=6)	-	1.2 (0.7-4.8, n=7)	2.8 (1.7-4.3, n=4)	1.7 (0.8-3.9, n=5)
T=48h (E29 + 120h[5])	2.8 (2.3-3.3, n=2)	-	1.7 (0.9-2.3, n=4)	2.9 (1.3-3.7, n=3)	3.0 (2.5-4.4, n=4)

[1]rhIGF-1/rhIGFBP-3 was administered at approx. 72 hours of age.

[2]Sample was obtained at termination, i.e. one liver mRNA IGF-1 value at one timepoint was obtained per animal. Timepoint is presented as time after rhIGF-1/rhIGFBP-3 administration (corresponding age of pup at sampling/termination).

[3]Data is presented as median fold change (range, n) vs. expression levels at T=0h (E29 + 0h). Data was normalized to ACTB.

[4]Differences between vehicle treated pups vs. rhIGF-1/rhIGFBP-3 treated pups at respective time-points were analyzed using one-way ANOVA with post hoc Bonferroni. *$P<0.05$.

[5]Samples collected at approx. 96-120 hours of age.

n = Number of pups, h = hours, mg = milligram, kg = kilogram.

Supplementary Table 6. Body weight following exposure to rhIGF-1/rhIGFBP-3[1]

Timepoint[2]	Vehicle (g)[3]	1.0 mg/kg rhIGF-1/rhIGFBP-3 (g)[3]	2.0 mg/kg rhIGF-1/rhIGFBP-3 (g)[3]	4.0 mg/kg rhIGF-1/rhIGFBP-3 (g)[3]	8.0 mg/kg rhIGF-1/rhIGFBP-3 (g)[3]
T=0h[6] (E29 + 0h)	40 (33-49, n=14)	-	-	-	38*[5] (24-42, n=13)
T=24h (E29 + 24h)	47*[4] (36-55, n=8)	-	-	-	45 (37-59, n=10)
T=48h (E29 + 48h)	45 (30-54, n=10)	-	-	-	46 (35-56, n=9)
T=72h[7] (E29 + 72h)	44 (36-54, n=10)	49 (38-50, n=10)	42 (30-53, n=25)	35*[5] (27-49, n=6)	46 (30-61, n=18)
T=96h (E29 + 96h)	-	-	40 (35-55, n=7)	44 (39-58, n=7)	39 (24-50, n=20)
T=120h (E29 + 120h)	-	-	43 (34-47, n=7)	37 (34-41, n=3)	44 (40-48, n=4)

[1]rhIGF-1/rhIGFBP-3 was administered at approx. 72 hours of age for all groups except 8.0 mg/kg sampled at T=0, T=24, and T=48h, in which rhIGF-1/rhIGFBP-3 was administered at approx. 3 hours or age.

[2]Sample was obtained at termination, i.e. one serum IGF-1 value at one timepoint was obtained per animal.

[3]Data is presented as median (range, n).

[4]Differences between vehicle treated pups at T=0h (E29 + 0h) vs. vehicle treated pups at respective time-points were analyzed using one-way ANOVA with post hoc Bonferroni. *$P<0.05$, **$P<0.01$, ***$P<0.001$.

[5]Differences between vehicle treated pups vs. rhIGF-1/rhIGFBP-3 treated pups at respective time-points were analyzed using one-way ANOVA with post hoc Bonferroni. *$P<0.05$, **$P<0.01$, ***$P<0.001$.

[6]Samples collected at approx. 7-15 hours of age.

[7]Samples collected at approx. 72-80 hours of age.

n = Number of pups, h = hours, mg = milligram, kg = kilogram, g = gram

Supplementary Figure 1

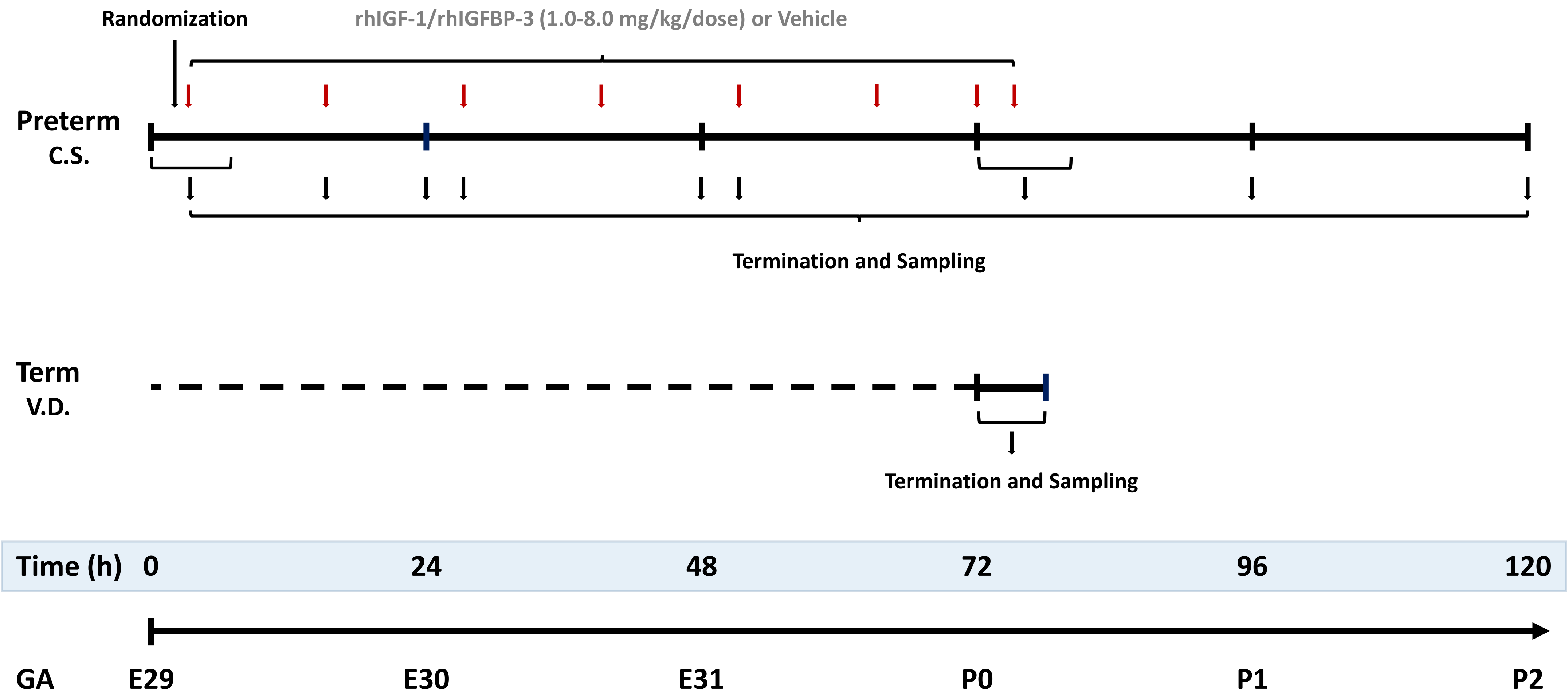

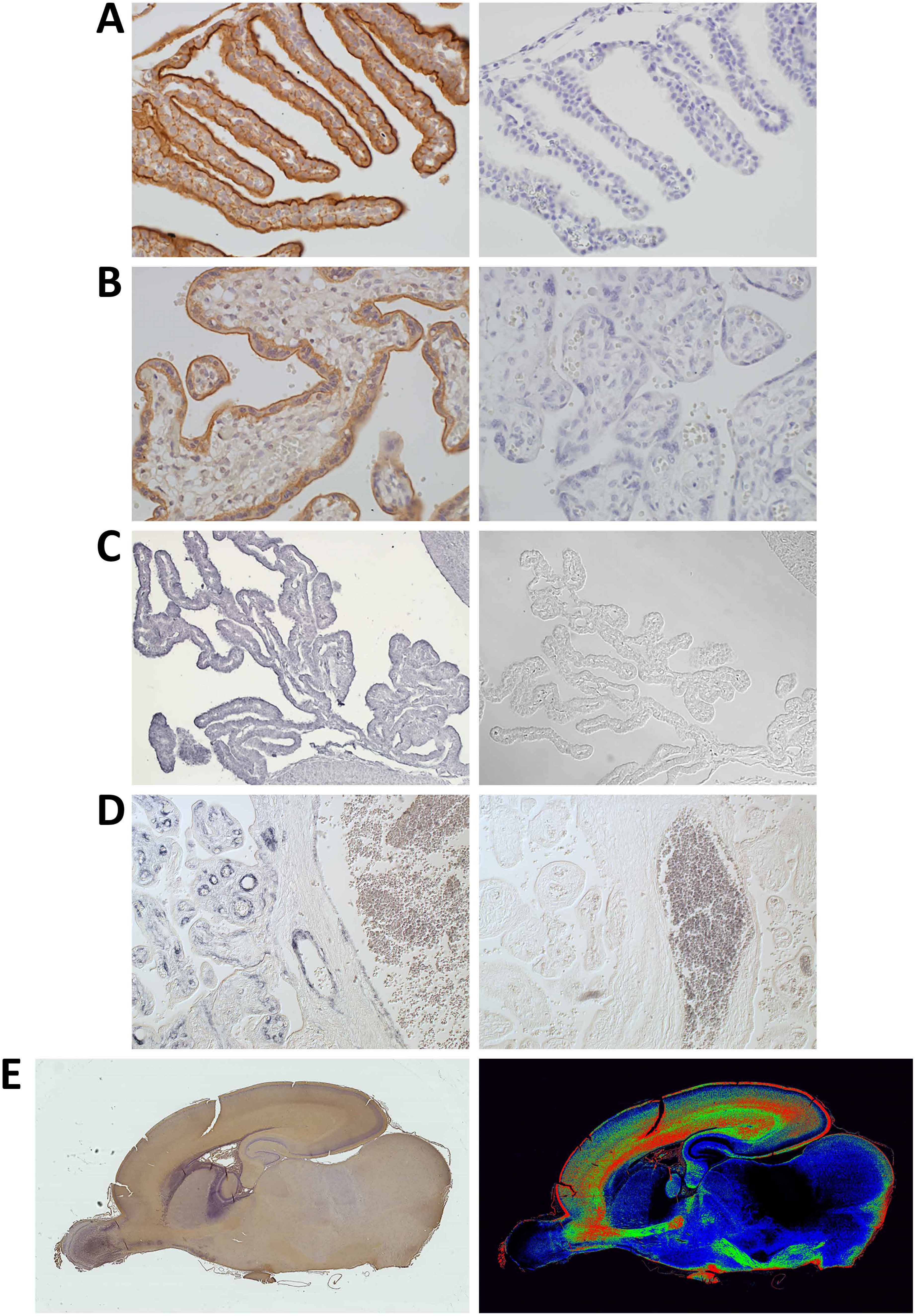
A
B
C
D
E
